# Histotripsy-induced vascular remodeling in the tumor microenvironment enhances local and off-target intratumoral drug delivery

**DOI:** 10.64898/2026.09.18.752667

**Published:** 2026-09-24

**Authors:** Heineken Queen, Brian Song, Hanna Kim, Reliza McGinnis, Jiayu Liu, Aichata Sanogo, Katherine Buglak, Chaitanya Karanam, Allison Bontrager, Tejaswi Worlikar, Zhen Xu, Anutosh Ganguly, Clifford S. Cho

## Abstract

Solid tumors are known to be refractory to treatment due to a poorly perfused microenvironment that blocks immune cell infiltration and effective drug delivery. Histotripsy is a non-invasive focused ultrasound modality that generates cavitation microbubbles to mechanically disrupt solid tumors, resulting in decreased hypoxia and stimulation of tumor-targeted adaptive immune responses. In this study, we aim to investigate whether post-histotripsy hypoxia reduction and cytotoxic immune cell infiltration are associated with normalization of tumor vasculature and enhanced perfusion. Structural analysis of both treated and contralateral off-target tumors following partial histotripsy revealed significant vascular remodeling, enhanced vascular integrity, and decreased vascular leakage indicative of vascular normalization in multiple *in vivo* models of melanoma, hepatocellular carcinoma, and pancreatic adenocarcinoma. These effects appeared antigen-specific, as no remodeling was observed in contralateral off-target tumors of discordant pathology. Mechanistically, CXCR3+CD8+ T cells played a role in mediating these vascular changes, as genetic knockout of CD8 and pharmacological antagonism of CXCR3 impaired both remodeling and normalization. Additionally, significant changes in endothelial CXCR4 and Angiopoietin-1 and -2 expressions were observed following histotripsy, indicating their potential involvement in promoting vascular normalization and remodeling. Significant vascular remodeling was accompanied by enhanced tumor perfusion and intratumoral delivery of chemotherapeutic agents and therapeutic monoclonal antibodies. These findings suggest that histotripsy not only creates a more favorable microenvironment for immunotherapy, but also optimizes drug delivery into solid tumors. Combinatorial approaches using histotripsy with chemotherapy or immunotherapy may be a novel therapeutic strategy to overcome current limitations of conventional solid tumor therapies.

**Summary:**

Histotripsy reorganizes the tumor vasculature of solid cancers, promoting enhanced drug delivery into treated and non-targeted distant tumors.

## Full Text Availability

The license terms selected by the author(s) for this preprint version do not permit archiving in PMC. The full text is available from the preprint server.

